# Post-execution monitoring in dishonesty

**DOI:** 10.1007/s00426-022-01691-x

**Published:** 2022-06-25

**Authors:** Anna Foerster, Roland Pfister, Robert Wirth, Wilfried Kunde

**Affiliations:** Department of Psychology III, University of Würzburg, Röntgenring 11, 97070 Würzburg, Germany

## Abstract

When telling a lie, humans might engage in stronger monitoring of their behavior than when telling the truth. Initial evidence has indeed pointed towards a stronger recruitment of capacity-limited monitoring processes in dishonest than honest responding, conceivably resulting from the necessity to overcome automatic tendencies to respond honestly. Previous results suggested monitoring to be confined to response execution, however, whereas the current study goes beyond these findings by specifically probing for post-execution monitoring. Participants responded (dis)honestly to simple yes/no questions in a first task and switched to an unrelated second task after a response–stimulus interval of 0 ms or 1000 ms. Dishonest responses did not only prolong response times in Task 1, but also in Task 2 with a short response–stimulus interval. These findings support the assumption that increased monitoring for dishonest responses extends beyond mere response execution, a mechanism that is possibly tuned to assess the successful completion of a dishonest act.

**Supplementary Information:**

The online version contains supplementary material available at 10.1007/s00426-022-01691-x.

## Introduction

Our knowledge of the cognitive processes underlying dishonest responding has been steadily progressing over the last decades. Apart from demonstrating that responding dishonestly is demanding in general (e.g., Zuckerman et al., 1981), multiple findings have suggested that dishonest responding, or lying, comes with specific peculiarities as compared to honest responding (e.g., Vrij et al., 2008; Walczyk et al., 2014). One such supposed peculiarity is that liars engage more effort to monitor their own behavior, as well as the behavior of the recipient. Recent empirical work on the cognitive foundations of these monitoring processes has indeed yielded evidence for an increased recruitment of capacity-limited monitoring, at least for the duration of one’s own response (Foerster et al., 2019). The current study addresses monitoring processes that outlast response execution.

### Sources of monitoring

Agents seem to dedicate considerable effort into monitoring the success of *any* of their responses (e.g., Jentzsch et al., 2007; Welford, 1952) though monitoring is likely to be especially pronounced for dishonest responses. An elaborate theory on lying, the Action–Decision–Construction–Action Theory (ADCAT), proposes that monitoring of dishonest responses serves two purposes: ensuring that own behavior unfolds as intended and inferring the believability of the lie from the recipient’s behavior (Walczyk et al., 2014). Whereas monitoring of the persuasiveness of a lie might be a deliberate, motivated process, the former type of monitoring may emerge automatically as a direct and immediate consequence of the cognitive operations mediating dishonest behavior. These cognitive operations are usually studied in highly controlled paradigms, where participants are prompted to respond honestly and dishonestly to simple questions as fast and accurately as possible via keypresses (e.g., Furedy, et al., 1988; Suchotzki et al., 2017).

To understand why monitoring could differ in extent between dishonest and honest responses, we propose to consider the underlying conflicting nature of dishonesty. Delivering a dishonest response comes with the obstacle of overcoming the initially, automatically activated truthful response in most situations (e.g., Duran et al., 2010; Walczyk et al., 2003). It seems that this initial activation of the truthful response is not only an unwelcome side effect, but rather an integral part of cognitive processing during dishonesty: The presentation of honest rather than dishonest response options as irrelevant distractors has been shown to facilitate honest and dishonest responding alike (Debey et al., 2014; Foerster et al., 2017a, 2017b). The activation of competing action plans is the defining feature of cognitive conflict which, in turn, activates cognitive control processes (e.g., Botvinick et al., 2001). According to conflict-monitoring theory, the detection of any conflict should thus trigger control adjustments to avoid or deal with similar conflicts in the future. Crucially, cognitive conflict is a necessary by-product of responding dishonestly in the face of an automatically activated truthful response, whereas honest responding does not come with a similar degree of conflict.

This perspective highlights striking similarities of honest vs. dishonest responding with the comparison of cognitive consequences of error commission: Committing an error should elicit a conflict between the intended and the delivered response, whereas no such conflict arises for typical correct responses. Because errors are known to trigger monitoring processes as a direct consequence of this conflict (accompanied by additional post-error adjustments; Jentzsch, & Dudschig, 2009; Steinhauser et al., 2017), it seems plausible to assume structural similarities for dishonesty (Foerster et al., 2018, 2019). That is, both dishonest responses and errors come with the parallel activation of competing action tendencies and the resulting conflict may trigger monitoring. These monitoring processes assess whether a response unfolds as intended, and they may lead to control adaptations that aim for more efficient and accurate (dishonest) responses in the future.

### Localizing response monitoring

While response monitoring has been considered an integral part of information processing (Welford, 1952), little research has addressed the question which information processing stages are concerned with such monitoring (e.g., Pashler, 1994). Classical stage theory confines the processing between stimulus presentation and response execution, dividing it into a precentral, a central and a postcentral stage. The precentral and postcentral stages map mostly to perceptual and motor processes, respectively, and can operate in parallel with processing stages of another task. In contrast, the central stage is assumed to be capacity-limited and, amongst other things, devoted to response selection (for more fine-grained analyses, see Hommel, 1998; Janczyk et al., 2014). As monitoring is often proposed to be capacity-limited (Jentzsch & Dudschig, 2009; Jentzsch et al., 2007), central processes of another task should not be able to operate in parallel with ongoing monitoring.

Evidence for capacity-limited monitoring during dishonest responding comes from psychological refractory period methodology (Experiment 3 in Foerster et al., 2019). Participants responded honestly and dishonestly to yes/no questions about daily activities in a first task, and to the pitch of a tone in a second task, with a short or long stimulus-onset asynchrony between question and tone. The assumption that dishonest responding requires a longer central processing stage than honest responding predicts that the resulting response time (RT) costs in Task 1 could also affect processing of the nominally unrelated Task 2 by postponing its capacity-limited central stage (see Foerster et al., 2019, for a detailed argument). This was indeed the case, and this finding is consistent with the idea that overcoming the activated truthful response itself is capacity-limited. Crucially, the RT costs for dishonesty were even larger in Task 2 than in Task 1 if both tasks occurred in close succession (i.e., at a short stimulus-onset asynchrony). This latter finding seems to suggest that capacity-limited monitoring may be recruited in addition to the costs of overcoming the automatically activated truthful response in Task 1.

**Fig. 1:**
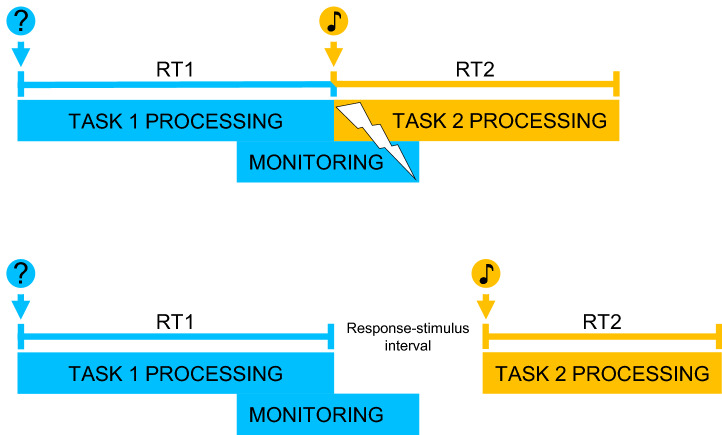
Illustration of the idea that monitoring processes exceed responding of a Task 1 and disturb information processing of Task 2. Responding to a question in Task 1 should trigger such monitoring, but importantly, dishonest responses should engage the monitoring process longer than honest responses. If a different, unrelated Task 2 follows the Task 1 response immediately rather than after a delay, monitoring should interfere with its processing, prolonging RT2

The described experimental setup with temporally overlapping tasks may reveal monitoring costs, but it cannot assess whether the assumed processes are still operating after response execution. The same pattern of results would emerge if either monitoring accompanied the postcentral stage of Task 1 but terminated at the time of responding, or if monitoring exceeded response execution. Here we propose to adopt a critical change in the experimental paradigm to distinguish between both theoretical possibilities: Presenting the same two tasks without temporal overlap, but rather in close temporal succession (see Fig. 1 for an illustration). Such paradigms delivered evidence for increased capacity-limited monitoring of erroneous responses after response execution (Jentzsch & Dudschig, 2009), whereas no such findings of prolonged monitoring emerged for dishonest responding (Experiment 4 in Foerster et al., 2019).

To conclude, tried and tested stage-oriented approaches allowed to examine monitoring of dishonest responding. Initial evidence from paradigms with overlapping tasks supports the notion of temporally extended monitoring processes with dishonest compared to honest responding but does not inform about the extent and point in time of these processes. Specifically, whether monitoring exceeds response execution can be investigated when tasks are presented in succession, but a first attempt did not show any differences in monitoring between honest and dishonest responding (Foerster et al., 2019).

### The present experiments

Why did previous research fail to demonstrate extended monitoring of dishonest responses after response execution? A first possibility is that monitoring of dishonesty indeed terminates with response execution. Second, participants received explicit accuracy feedback in Task 1 in previous studies (Foerster et al., 2019). In case of a Task 1 error, no stimulus for Task 2 appeared, but a new trial started. The stimulus onset of Task 2 thus indicated that (dis)honest responding was successful and this feedback signal might have aborted any monitoring processes to determine the success of the response. Third, the monitoring process itself might be relatively short-lived. If it did exceed responding in Task 1 but not the precentral processing of Task 2, it could not have delayed the subsequent capacity-limited central processing of Task 2 either, and thus could not have showed up in the RT of Task 2.

We addressed these alternative explanations in the current set of experiments to assess whether monitoring proceeds after delivering a dishonest response. To enable a conservative test of this hypothesis, we did not introduce any requirements for monitoring serious consequences of dishonest behavior, but rather aimed at removing factors that might have actively worked against monitoring tendencies in our previous work. Experiment 1 thus examined whether the presentation of error feedback counters post-response monitoring. In Experiment 2, we changed Task 2, intending to reduce precentral processing for it, and thus increase the chance to reveal capacity-limited monitoring costs. Experiment 3 delivered a high-powered replication of post-response monitoring costs. We preregistered hypotheses, sample sizes, exclusion criteria and statistical analyses of all three experiments (Experiment 1: osf.io/5c8jk, Experiment 2: osf.io/bm73c, Experiment 3: osf.io/z4ecx). These preregistrations, as well as materials, the collected data, and analyses scripts are publicly accessible on the *Open Science Framework* (osf.io/7axw9).

## Experiment 1

We probed whether monitoring of dishonest responses terminates with response execution or whether it outlasts response execution. Following the idea that certainty about the success of the (dis)honest response might diminish monitoring, we manipulated the time point of error feedback of (dis)honest responses in a Task 1, which appeared either early, before the Task 2 stimulus, or late, after the Task 2 response.

In all other aspects, the current experiment replicated the already published experiment from our lab (Experiment 4 in Foerster et al., 2019). We employed an Intention Task 1, where participants responded honestly and dishonestly to yes/no questions about daily activities and a Tone Task 2, where the participants classified the pitch of a tone. The response–stimulus interval between the two tasks was 0 ms or 1000 ms.

We employed a paradigm that we assume to differentiate between honest and dishonest responding mostly concerning the presence of cognitive conflict while controlling for any other differences (Furedy et al., 1988). We expected prolonged RTs and higher error rates for dishonest compared to honest responses in the Intention Task 1. We expected the intention effect in RTs to propagate to the Tone Task 2 with a response–stimulus interval of 0 ms and late error feedback. (Dis)honesty should have a smaller or even no impact in the remaining conditions, i.e., at a response–stimulus interval of a 1000 ms or with early error feedback.

### Methods

#### Participants

We could not rely on an observed effect size for the hypothesized modulation. We thus decided to base our power analysis for modulations of the effect of (dis)honesty by response–stimulus and error feedback on a hypothetical *d*_*z*_ = 0.5. Thirty-two participants provide a power of 80% to detect that effect size in a two-sided test with an alpha of 5% (calculated with the power.t.test function in R version 3.3.3).

We collected data of 36 participants (mean age = 28 [SD = 10.02] years), because four participants did not meet the preregistered inclusion criteria and had to be replaced: They responded correctly in less than 60% of experimental trials. Eight participants self-identified as male and three as left-handed. All participants provided written informed consent and received monetary compensation or course credit.

#### Apparatus and stimuli

We conducted the experiment in sessions with up to five participants in separate cubicles. Participants worked on computers with 24′′ screens, display resolutions of 1920 × 1080 and refresh rates of 100 Hz. They responded with their left and right index and middle fingers on a QWERTZ keyboard. The Intention Task 1 required responding with *yes* and *no* to questions about daily activities via the keys *D* and *F.* Table 1 in Appendix A lists the whole question pool that we adapted from previous work (Foerster et al., 2019, 2017a, 2017b; Van Bockstaele et al., 2012). The color of the question, blue and yellow, indicated to respond honestly or dishonestly. The Tone Task 2 required categorizing a 300 Hz and an 800 Hz tone of 100 ms length as low or high via the keys *K* and *L*. Participants heard these tones via headphones. We counterbalanced the assignment of responses to keys and colors to intentions.

#### Procedure

The selection of a question set preceded the actual experimental procedure. Participants responded with *yes* and *no* via the appropriate keypress and had to indicate whether they had or had not performed each inquired activity at the day of the experiment for a random subset of the 72 questions. Instructions strongly emphasized the importance to provide correct responses in this phase and to discuss any uncertainties or errors with the experimenter. The procedure stopped when participants had honestly affirmed and negated ten questions, respectively. The experiment proceeded only with the first ten affirmed and first ten negated questions, discarding any surplus questions.

Participants received instructions about the gist of the two tasks. They then first learned about the assignment of colors to honest and dishonest responding and went through a practice block of eight trials with the Intention Task 1 only. They responded honestly and dishonestly to two practice questions (i.e., “Are you at the beach?” and “Are you in a room”) in this block. Afterward, they went through eight practice trials of the Tone Task 2 only. In all practice trials, participants could select their response without any time restrictions. Specific error feedback appeared in case of an early response before stimulus onset or a false response.

Experimental trials commenced with a blank screen of 500 ms, followed by a fixation cross of the same duration. Then the colored question appeared in the center of the screen, requiring an honest or dishonest response. The response labels *yes* and *no* appeared on the left and right, according to key assignment, in the lower half of the screen as a reminder. The display cleared after a correct response or after 3000 ms elapsed. In half of the trials, a response–stimulus interval of 1000 ms preceded the tone, in the other half, the tone followed immediately after the response to the Intention Task 1. Participants had to respond within 1000 ms after tone onset.

We provided error-specific feedback in red font for 1500 ms and manipulated the moment of its presentation for errors in the Intention Task 1. Omitting a response or committing a false response in the Intention Task 1 triggered feedback either early, i.e., immediately after the incorrect response, or late, i.e., after responding to the tone. In either case, participants conducted the Tone Task 2. Feedback for similar errors in the Tone Task 2 always appeared immediately after completing this task. Omission errors were fed back with “Too late” and commission errors with “False” (German: “Zu spät” and “Falsch”). In the early and the late feedback condition alike, feedback for Task 1 was presented slightly above the center of the screen and feedback for Task 2 in the same distance from the center, but in the lower part, of the screen. In addition, the error message always included the task, e.g., “Question: False” or “Tone: Too late”. If one of the two tasks was erroneous and the other one correct in the late feedback condition, feedback for the correct response was presented in green font, e.g., “Tone: OK”. Early responses during the blank screen, fixation or during the response–stimulus interval immediately aborted the trial and led to the error feedback “Too early” (German: “Zu früh”).

The combination of 20 questions × 2 intentions (honest vs. dishonest) × 2 response–stimulus intervals (0 ms vs. 1000 ms) × 2 tones (300 Hz vs. 800 Hz) led to 160 individual trials in a random sequence within each block. Participants conducted four blocks with self-paced breaks after each 40th trial. In two successive blocks, participants received error feedback for the Intention Task 1 early, while in two other successive blocks, they received it late. We counterbalanced the order of early and late error feedback across participants.

### Results

#### Data treatment

We excluded practice trials. We then eliminated the first trial after each break and selected only post-correct trials (20.6% excluded). To analyze the error rates of the Intention Task 1, we excluded premature responses (0.1%) and omission errors (0.6%). For the error rate analysis of the Tone Task 2, we selected trials with a preceding correct (dis)honest response and eliminated premature responses (0.4%) and omission errors (2.1%) of the Tone Task 2. For RT analyses of both tasks, we selected trials with only correct responses and eliminated trials with RTs that deviated more than 2.5 standard deviations from their respective cell mean as outliers (4.4%). All participants delivered at least 10 observations in each cell after these exclusions and could thus be included in the following statistical analyses.

#### Analyses plan

**Fig. 2:**
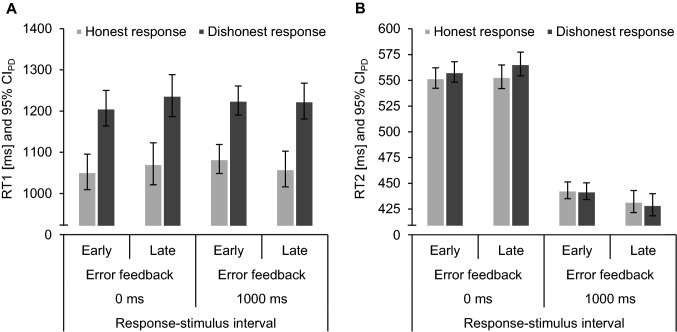
Mean response times (RTs) of the Intention Task 1 (**A**) and the Tone Task 2 (**B**) of honest (light grey) and dishonest (dark grey) responses in Experiment 1. Error bars depict 95% confidence intervals of paired differences (CI_PD_; Pfister & Janczyk, 2013), computed separately for each error feedback and response–stimulus interval

Tables 3 and 4 in Appendix B give an overview of the descriptive statistics. Figure 2 depicts mean RTs. We analyzed error rates and RTs in analyses of variance (ANOVAs) with the within-subjects factors error feedback (early vs. late), intention (honest vs. dishonest) and response–stimulus interval (0 ms vs. 1000 ms). Note that error feedback did not appear for any trials of the RT analysis (as only correct trials were included in this analysis), whereas the factor error feedback codes how error feedback was presented in the current block. We scrutinized significant three-way interactions in planned 2 × 2 ANOVAs for each response–stimulus interval and significant two-way interactions in planned two-tailed paired-samples *t*-tests.

#### Intention task (Task 1)

Dishonest responses were slower than honest responses, *F*(1, 31) = 77.75, *p* < 0.001, *η*_p_^2^ = 0.72, whereas the remaining main effects were not significant, *F*s ≤ 1.18, *p*s ≥ 0.286, *η*_p_^2^ ≤ 0.04. A significant interaction between response–stimulus interval and error feedback emerged, *F*(1, 31) = 8.95, *p* = 0.005, *η*_p_^2^ = 0.22. Other interactions were not significant, *F*s < 1. We averaged across intentions to scrutinize the significant two-way interaction. For the response–stimulus interval of 0 ms, participants responded descriptively faster with early compared to late error feedback, *t*(31) = −2.79, *p* = 0.009, *d*_*z*_ = −0.49. In contrast, responses were descriptively slower with early than late feedback for the response–stimulus interval of 1000 ms, *t*(31) = 1.64, *p* = 0.111, *d*_*z*_ = 0.29.

Error rates increased for dishonest compared to honest responses, *F*(1, 31) = 60.67, *p* < 0.001, *η*_p_^2^ = 0.66, whereas the remaining main effects and all two-way interactions were not significant, *F*s ≤ 2.10, *p*s ≥ 0.158, *η*_p_^2^ ≤ 0.06. The three-way interaction was significant, *F*(1, 31) = 6.30, *p* = 0.018, *η*_p_^2^ = 0.17. Separate ANOVAs indicated that intention and feedback arrangement interacted for the response–stimulus interval of 1000 ms, *F*(1, 31) = 5.20, *p* = 0.030, η_p_^2^ = 0.14, but not for the interval of 0 ms, *F*(1, 31) < 1. For the response–stimulus interval of 1000 ms, the difference between dishonest and honest responses was larger with early feedback, *t*(31) = 6.30, *p* < 0.001, *d*_*z*_ = 1.11, than with late feedback, *t*(31) = 4.19, *p* < 0.001, *d*_*z*_ = 0.74.

#### Tone task (Task 2)

Shorter RTs emerged with a response–stimulus interval of 1000 ms compared to 0 ms, *F*(1, 31) = 178.68, *p* < 0.001, η_p_^2^ = 0.85. Other main effects were not significant, *F*s ≤ 1.89, *p*s ≥ 0.179, η_p_^2^ ≤ 0.06. However, the response–stimulus interval modulated the effect of intention, *F*(1, 31) = 6.62, *p* = 0.015, *η*_p_^2^ = 0.18. The interaction of response–stimulus interval and error feedback was slightly above the significance level, *F*(1, 31) = 3.65, *p* = 0.065, *η*_p_^2^ = 0.11, and other interactions were not significant either, *F*s < 1. We averaged across error feedback to scrutinize the significant two-way interaction and observed that preceding dishonest compared to honest responses prolonged RT2 after a response–stimulus interval of 0 ms, *t*(31) = 2.36, *p* = 0.025, *d*_*z*_ = 0.42, but not after 1000 ms, *t*(31) = −0.68, *p* = 0.501, *d*_*z*_ = −0.12.

An increase in response–stimulus interval resulted in lower error rates, *F*(1, 31) = 26.16, *p* < 0.001, *η*_p_^2^ = 0.46. The analysis of error rates indicated no other significant main effects or interactions, *F*s ≤ 2.23, *p*s ≥ 0.146, *η*_p_^2^ ≤ 0.07.

### Discussion

The current experiment investigated whether dishonest responses are monitored more extensively than honest responses even after response execution has already finished. We assessed monitoring of (dis)honest responses of an Intention Task 1 via RTs in an unrelated Tone Task 2. We expected RT2 to be longer after dishonest than honest responses if Task 2 succeeded promptly after responding in Task 1 in blocks with relative uncertainty about response success in Task 1.

The obtained evidence is in line with the assumption of increased monitoring of dishonest responses after response execution. Dishonest responses were not only slower and more error-prone than honest responses in the Intention Task 1, but this influence propagated to responding in the Tone Task 2 at the short response–stimulus interval. This qualifies past results, which suggested the existence of capacity-limited monitoring of dishonest responses, but which did not support the notion of monitoring after response execution (Foerster et al., 2019).

A modulation of post-response monitoring by response uncertainty in the current experiment would have delivered a good reason for these diverging results. However, monitoring effects emerged equally in blocks with early and late error feedback for (dis)honest responses, although there was a descriptive trend toward smaller monitoring costs with immediate than late feedback. For one, this might suggest that either the absent monitoring effects in the literature is a beta error (Exp. 4 in Foerster et al., 2019), or the significant monitoring effects here is the result of an alpha error. The highly powered replication of the monitoring costs in Experiment 3 argues strongly against an alpha error here.

Future studies on monitoring should expand on the role of response uncertainty for post-response monitoring in general. In the current study, we could not explore the impact of response uncertainty via the timepoint of error feedback on monitoring after errors, because the early feedback condition increased the response–stimulus interval between tasks by 1500 ms. Any monitoring effects should have been completed in this extended period and, therefore, before Task 2 commenced. In addition, monitoring costs after the short RSI were small in our study. To address both issues, researchers might modulate response uncertainty via other modalities than the one that both tasks are presented in, for example, using keys that randomly do or do not move when being pressed. Such a manipulation of tactile instead of visual feedback would allow for an immediate presentation of Task 2 stimuli after responding to Task 1, enabling the assessment of monitoring effects. Furthermore, minimized proximal feedback by removing tactile response feedback, might boost monitoring processes. Finally, larger sample sizes would capture potentially smaller effects of response uncertainty on monitoring.

Although monitoring costs can become evident in error rates (Jentzsch & Dudschig, 2009; Steinhauser et al., 2017), they did not emerge here, nor in preceding investigations on dishonesty (Experiment 3 in Foerster et al., 2019). The absence of an effect in all these studies could be due to a floor effect, because error rates in Task 2 were consistently low. In any case, the resulting error rates do not suggest a speed–accuracy trade-off at the heart of monitoring effects.

Responses in the Tone Task 2 became more efficient with an increase in response–stimulus interval. These results were expected but not informative about our hypothesis. We had no predictions about the observed interactions in error rates and RTs of the Intention Task 1. Because these seem unproblematic for our hypothesized effects in the Tone Task 2, we would like to refrain from interpreting them.

## Experiment 2

Experiment 2 was a conceptual replication of Experiment 1. As we did not find a modulation by the time of error feedback presentation (early vs. late), we decided to focus on demonstrating post-execution monitoring costs using only the more promising condition with late error feedback here. Furthermore, we aimed for a stronger propagation of monitoring effects from Task 1 to Task 2 by having participants respond to two letters instead of tones in Task 2. This allowed participants to direct attention to task-relevant stimuli in only one rather than two different perceptual modalities. Moreover, visual stimuli have a more clearly defined onset than auditory stimuli, which conceivably reduces any technically caused variability between responding in Task 1 and the presentation of the stimulus for Task 2, thereby increasing the chances to reveal statistically robust monitoring costs. We also expected shorter precentral processing of visual than auditory stimuli but evidence from the literature about this aspect is mixed (e.g., Robinson et al., 2018; Spence et al., 2012). Finally, we intended to assess honest and dishonest responding in a more controlled approach. In Experiment 1, participants responded to different selections of questions depending on their activities outside the laboratory on the day of data collection. In Experiment 2, we instead introduced a highly controlled question set in in the Intention Task 1, where we asked all participants about the same activities they did or did not perform in the laboratory (see also Experiments 1 and 2 in Foerster et al., 2019).

We again expected prolonged RTs and higher error rates for dishonest compared to honest responses in the Intention Task 1. We also expected an intention effect in RTs of the Letter Task 2 with a response–stimulus interval of 0 ms but not with a response–stimulus interval of 1000 ms.

### Methods

We keep this section brief by reporting only aspects, where Experiment 2 deviated from Experiment 1.

#### Participants

The modulation of RTs of the Tone Task 2 by intention and response–stimulus interval amounted to *d*_*z*_ = 0.45 (${d}_{z}= \frac{t}{\sqrt{n}}= \frac{\sqrt{F}}{\sqrt{n}}= \frac{\sqrt{6.62}}{\sqrt{32}}$) in Experiment 1. We expected to find this two-way interaction in a similar size in Experiment 2 and, therefore, used this effect size for the power analysis. Considering our counterbalancing factors, we decided to include 48 participants in our statistical analyses. A sample size of 48 participants provides a power of 86% to detect an effect of this size in a two-sided test with an alpha of 5% (calculated with the power.t.test function in R version 3.3.3). Note that we expected a stronger modulation in the Letter Task 2 (compared to the Tone Task 2 used in Experiment 1).

We had to collect data of 53 participants (mean age = 27 [SD = 8.61] years), because five participants did not qualify for inclusion as in Experiment 1 and required replacement. Fifteen participants self-identified as male and one as non-binary. Nine participants reported to be left-handed.

#### Apparatus and stimuli

Participants conducted a set of 10 out of 20 simple activities in the laboratory, e.g., they detached a wire from a cap (see Experiment 2 in Foerster et al., 2019, for a similar procedure) and we counterbalanced the two sets of activities across participants. The Intention Task 1 featured 20 questions about the performance of each activity (see Table 2 in Appendix A), e.g. “Did you detach the wire from the cap?”. As such, each participant gave affirmative responses to one half of the questions and negative responses to the other half of the questions to be honest and vice versa to be dishonest.

In the Letter Task 2, participants categorized the letters *H* and *S* via the comma and dot response keys on a QWERTZ keyboard. We counterbalanced the assignment of letters to keys across participants.

#### Procedure

The experimenter prepared boxes with the necessary objects to perform one set of activities. Participants within the same session performed the same set of activities to exclude any input from not performed activities. Participants received separate instructions to perform each of the ten activities carefully on screen. They could proceed from the instruction of one activity to the next by keypress after a forced break of 5 s. The experimenter checked whether participants had performed each activity correctly. If necessary, the experimenter repeated the instructions of activities if there was any mistake until participants had performed all instructed activities correctly.

Feedback for omission and commission errors in the Intention Task 1 appeared always after responding to the Letter Task 2. The combination of 20 questions × 2 intentions (honest vs. dishonest) × 2 response–stimulus intervals (0 ms vs. 1000 ms) × 2 letters (H vs. S) resulted in 160 different trials. Participants went through two blocks of these randomized trials with self-paced breaks after each 40th trial.

### Results

#### Data treatment

We applied the same exclusion criteria as in Experiment 1. We excluded practice trials, each trial that followed a self-paced break or an incorrect trial (17.2% excluded). We excluded premature responses (0.1%) and omission errors (0.8%) in the Intention Task 1 to analyze its error rates. To analyze error rates of the Letter Task 2, we excluded trials with an erroneous response in the Intention Task 1 or a premature response (0.1%) or omission error (1.8%) in the Letter Task 2. We excluded all trials with an error or outlier RT in any of the two tasks for RT analyses (4.1%). All participants delivered at least 10 observations in each cell after these exclusions and could thus be included in the following statistical analyses.

#### Analyses plan

**Fig. 3:**
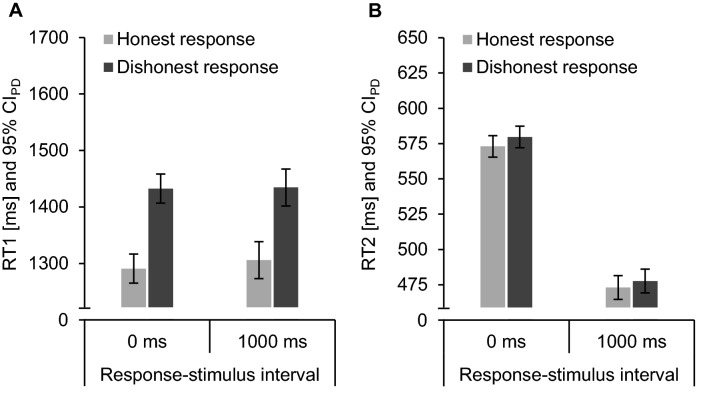
Mean response times (RTs) of the Intention Task 1 (**A**) and the Letter Task 2 (**B**) of honest (light grey) and dishonest (dark grey) responses in Experiment 2. Error bars depict 95% confidence intervals of paired differences (CI_PD_), computed separately for each response–stimulus interval

Detailed descriptive statistics are provided in Tables 5 and 6 in Appendix B and Fig. 3 illustrates mean RTs. Separate ANOVAs examined error rates and RTs for the within-subjects factors intention (honest vs. dishonest) and response–stimulus interval (0 ms vs. 1000 ms). We scrutinized significant two-way interactions in planned two-tailed paired-samples *t*-tests.

#### Intention task (Task 1)

Dishonest responses were slower, *F*(1, 47) = 117.25, *p* < 0.001, *η*_p_^2^ = 0.71, and more error-prone, *F*(1, 47) = 67.25, *p* < 0.001, *η*_p_^2^ = 0.59, than honest responses. Neither the main effect of response–stimulus interval nor the two-way interaction was significant in RTs, *F*s ≤ 1.29, *p*s ≥ 0.261, *η*_p_^2^ ≤ 0.03, or error rates, *F*s ≤ 1.68, *p*s ≥ 0.201, *η*_p_^2^ ≤ 0.04.

#### Letter task (Task 2)

A longer response–stimulus interval resulted in shorter RTs, *F*(1, 47) = 181.27, *p* < 0.001, *η*_p_^2^ = 0.79. Preceding dishonest responses prolonged RTs of the Letter Task 2 just descriptively, *F*(1, 47) = 4.05, *p* = 0.050, *η*_p_^2^ = 0.08, and the interaction of both factors was not significant, *F* < 1.

Error rates decreased with the response–stimulus interval of 1000 ms compared to 0 ms, *F*(1, 47) = 9.98, *p* = 0.003, *η*_p_^2^ = 0.18. Neither the main effect of intention nor the two-way interaction was significant, *F*s < 1.

### Discussion

Experiment 2 conceptually replicated Experiment 1. We expected prolonged responding in the Letter Task 2 after a dishonest compared to after an honest response in the Intention Task 1 with a response–stimulus interval of 0 ms, but not with an interval of 1000 ms. As expected, dishonest responses were slower and less accurate than honest responses in the Intention Task 1. This difference was neither convincingly present nor absent in the Letter Task 2 (*p* value equivalent to alpha) and further showed no modulation by response–stimulus interval.

A comparison of the mean RT of the tone (Experiment 1: M = 431 ms) and letter (Experiment 2: M = 476 ms) tasks for the late error feedback condition with a response–stimulus interval of 1000 ms suggests that we might have failed to find a robust monitoring effect, because we did not efficiently reduce precentral processing through using visual instead of auditory stimuli. Even though, mean response times represent the sum of precentral, central, and postcentral processes, this difference in RTs hints rather to an increase than to a reduction of the precentral stage.

The absent modulation of the elusive monitoring costs suggests that any potential aftereffects on the letter task might not only have been the result of early monitoring but also of late control adjustments (Jentzsch & Dudschig, 2009). Such a late mechanism of control adaptation and its transfer from (dis)honest responding to other (conflict) tasks needs further examination in the light of mixed results from this study (Experiments 1 vs. 2) and from published data (Foerster et al., 2017a, 2017b, 2018).

## Experiment 3

Experiment 3 aimed at a high-powered conceptual replication of monitoring costs for the conditions that boosted the chance of detecting monitoring in performance of Task 2 in the former experiments. Therefore, we resorted to tone stimuli for Task 2 as in Experiment 1. Feedback for incorrect (dis)honest responses always appeared late after responding in the Tone Task 2 as in Experiment 2. Furthermore, we kept the response–stimulus interval at 0 ms throughout the experiment and did not employ a response–stimulus interval of 1000 ms as in the former two experiments. Finally, we again employed the question and activity set in Task 1 as in Experiment 2 for a highly controlled assessment of honest and dishonest responding. We expected more incorrect and slower dishonest than honest responses in the Intention Task 1 and higher RTs after dishonest than honest responding also in the Tone Task 2.

### Methods

#### Participants

We keep this section brief by reporting only aspects, where Experiment 3 deviated from Experiments 1 and 2. We relied on the data of Experiment 1 for our power analysis and decided a-priori for a more efficient one-sided testing of our directed hypotheses. The modulation of RT2 by intention for trials with a response–stimulus interval of 0 ms and late error feedback amounted to *d*_*z*_ = 0.39. Considering counterbalancing factors, 64 participants ensure a power of 93% to detect this effect size in a one-sided test with an alpha of 5% (calculated with the power.t.test function in R version 3.3.3).

Even though we could not anticipate and thus preregister this exclusion, we had to replace four participants, because tones played via speakers instead of headphones on one computer. As such, these tones were audible for the other participants. The experimenter became aware of this violation of the experimental protocol only when the participants told her after finishing the experiment.

Apart from that, we had to collect data of 76 participants (mean age = 24 [SD = 5.52] years), because five participants did not qualify for the same inclusion criteria as in Experiments 1 and 2. Twelve identified as male and five participants reported to be left-handed.

#### Apparatus, stimuli, and procedure

We used the same laboratory activities and questions as in Experiment 2 (Table 2 in Appendix B). Feedback for omission and commission errors in the Intention Task 1 appeared only after responding to the Tone Task 2 and the response–stimulus interval was always 0 ms. The combination of 20 questions × 2 intentions (honest vs. dishonest) × 2 tones (300 Hz vs. 800 Hz) resulted in 80 different trials. Participants went through four blocks of these randomized trials with self-paced breaks after each 40th trial.

### Results

#### Data treatment

We eliminated practice trials from the data. We excluded the first trial after each self-paced break and post-error trials (21.7%). Premature responses (0.2%) and omission errors (1.1%) in the Intention Task 1 did not enter the analysis of error rates of this task. We conducted the error rate analysis of the Tone Task 2 on trials with a correct response in Task 1 and eliminated response omissions (1.5%) in the Tone Task 2. For both RT analyses, we selected trials with correct responses in the two tasks and excluded trials, where at least one response qualified as an outlier (4.4%). All participants delivered at least 10 observations in each cell after these exclusions and could thus be included in the following statistical analyses.

#### Analyses plan

**Fig. 4:**
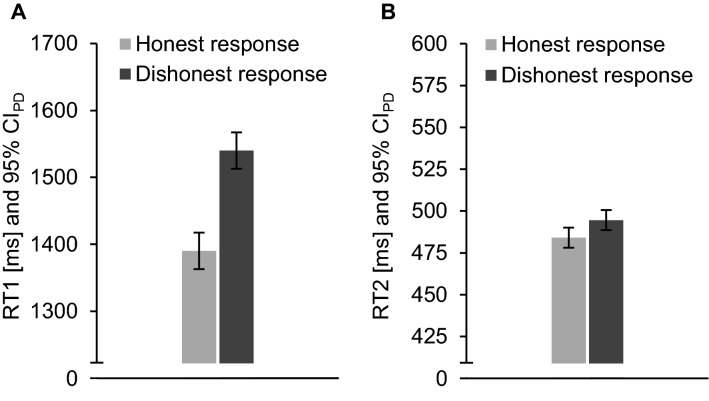
Mean response times (RTs) of the Intention Task 1 (**A**) and the Tone Task 2 (**B**) of honest (light grey) and dishonest (dark grey) responses in Experiment 3. Error bars depict 95% confidence intervals of paired differences (CI_PD_)

Tables 7 and 8 in Appendix B provide detailed descriptive statistics. Figure 4 shows mean RTs of both tasks. We examined whether dishonest responding increased error rates and RTs in one-tailed paired-samples *t*-tests.

#### Inferential statistics

Dishonest responses resulted in longer RTs, *t*(63) = 11.00, *p* < 0.001, *d*_*z*_ = 1.38, and more errors, *t*(63) = 10.44, *p* < 0.001, *d*_*z*_ = 1.30, than honest responses in the Intention Task 1. In the Tone Task 2, preceding dishonest responses increased RTs as compared to preceding honest responses, *t*(63) = 3.50, *p* < 0.001, *d*_*z*_ = 0.44, but did not affect error rates, *t*(63) = −0.13, *p* = 0.449, *d*_*z*_ = −0.02.

### Discussion

Experiment 3 was a high-powered replication of the experimental conditions that promised the highest likelihood to detect monitoring costs after response execution, and it did reveal such an effect. Taken together, we are confident to conclude that dishonest responses trigger monitoring even after response execution to a higher degree than honest responses.

## General discussion

The current set of experiments provides compelling evidence that dishonest responses are monitored more thoroughly than honest responses and that this monitoring process outlasts response execution. Previous evidence suggests that response-monitoring processes are capacity-limited, thereby delaying central processing of other tasks (Foerster et al., 2019; Jentzsch & Dudschig, 2009; Jentzsch et al., 2007; Steinhauser et al., 2017). The current results, therefore, enhance our understanding of the cognitive processes that render dishonest responding more demanding than honest responding. Whenever agents do not have a prepared or practiced lie at hand, an extended period of monitoring would allow them to keep track of the success of their response in being correct despite being confronted with conflicting behavioral tendencies. This demand entails incidental costs for subsequent unrelated behavior.

### The role of conflict

We proposed conflict between honest and dishonest response activation to be at the heart of the prolonged monitoring processes of dishonest responses, and we employed a paradigm that allowed for an isolated examination of this difference (Furedy et al., 1988). This close tie of cognitive conflict and monitoring would predict that a reduction in conflict, for example, through practice, preparation of false alibis or recent dishonest responding (Foerster et al., 2017a, 2017b; Hu et al., 2012; Suchotzki et al., 2018; Van Bockstaele et al., 2012), also diminishes monitoring costs. Whenever honest responses are more conflicting than dishonest responses, e.g., because dishonest responses had been practiced or prepared thoroughly, honest responses should trigger stronger monitoring than dishonest responses. In contrast, rendering dishonest response selection more difficult by having more than two response alternatives might boost monitoring of the success of dishonest responses. Such manipulations of the complexity of dishonest responding will, therefore, allow for assessing the role of monitoring for dishonesty more comprehensively.

On the other hand, responding is also more difficult in the presence of cognitive conflict leading to less accurate and slower dishonest performance (Suchotzki et al., 2017). As such, increased monitoring of dishonest responses could either be a specific consequence of cognitive conflict, or it could relate to response difficulty in general (Foerster et al., 2019). A potential first step to approach this issue in dishonesty could be to correlate monitoring costs with conflict strength, measured via a tried and tested distractor procedure (Debey et al., 2014; Foerster et al., 2017a, 2017b). Speculatively, agents might explicitly notice that they often struggle to respond efficiently and accurately when being dishonest and this knowledge might then boost monitoring. This perspective calls for an examination of the history of the success in responding honestly and dishonestly on monitoring. In any case, the current study points to a greater role of internal conflict and feedback processes than of the incorporation of explicit external feedback for the emergence of extended monitoring.

A further open issue pertains to the functional significance of post-response monitoring. One interpretation would be that these monitoring efforts still aim at ensuring that a response had been delivered as intended, assuming that such processing simply takes longer for dishonest responses than for honest responses, because more conflicting action tendencies emerge in action planning of dishonest responses. An alternative interpretation would hold that post-response monitoring rather aims at detecting additional, response-contingent changes in the environment. This latter interpretation receives support from studies on action–effect monitoring which indicate that monitoring extends to the immediate consequences of one’s own actions (Kunde et al., 2018; Wirth et al., 2018). Such a mechanism would allow for efficient detection of unintended consequences of one’s own responses, which are of particular relevance in case of dishonesty. Work on action–effect monitoring also indicates that the monitoring of own responses and monitoring of their effects seems to rely on common processes (Steinhauser et al., 2018). Whether and how such automatic monitoring processes interface with strategic and deliberate attempts at monitoring the behavior of recipients of one’s own lie (e.g., Walczyk et al., 2014) is an open question. In any case, the motivation to lie (believably) likely boosts monitoring processes (DePaulo et al., 1988; Porter & ten Brinke, 2010; Walczyk et al., 2014). Investigations about this relation should clearly distinguish between effects of motivation on response monitoring that are specific to dishonesty and effects that apply to more general behavior as well. Motivation might affect monitoring of dishonest responses directly if people assume that dishonest compared to honest responding changes their demeanor markedly and detectably or if they anticipate that the recipient of their lie critically assesses the truth value of their statements. An indirect link could be that highly motivated liars might experience less conflict and this, in turn, could reduce monitoring requirements.

Both proposed mechanisms might entail the assessment of the strength of conflict (i.e., between behavioral tendencies or between expected and encountered consequences) and this perspective suggests that monitoring might be a precursor for adaptation to dishonest responding. Akin to adaptation to other behavioral conflicts as in the Stroop or the Eriksen task, differences between dishonest and honest responding are modulated by (dis)honesty in the preceding action episode (Foerster et al., 2018). In particular, these differences vanished or reversed after a dishonest response, indicating a transition to a processing mode that facilitated overcoming the initial honest response tendency, rendering dishonest responses faster and less error-prone. Re-analyses of the current experiments replicated that effects of (dis)honesty in Task 1 were smaller after a dishonest response than after an honest response in Task 1 of the preceding trial, despite the intervening Task 2. Accordingly, the strength of conflict between honest and dishonest response tendencies might affect the extent of monitoring, and these monitoring processes might in turn trigger adaptation. Alternatively, conflict strength might trigger both, monitoring and adaptation, without a direct link between the latter to processes.

Apart from monitoring, aftereffects of dishonest responding on Task 2 might reflect alternative processes. For example, mutual inhibition between response alternatives in dishonest responding might also delay Task 2. If inhibition processes are indeed involved in dishonest responding, they should probably only target the initial representation of the honest response that has to be overcome (e.g., Walzyck et al., 2014); however, there is no empirical evidence for this claim, yet. To the contrary, boosting the activation of the honest response actually facilitates dishonesty (Debey et al., 2014; Foerster et al., 2017a, 2017b). Furthermore, Task 1 and Task 2 always employed different sets of response keys, which would call for inhibition of all motor activity rather than mutual inhibition of response alternatives to explain slowing in Task 2. In the case of errors, prolonged capacity-limited processes emerged even if response sets were different between Task 1 and Task 2 (Steinhauser et al., 2017). Although other researchers proposed orienting processes or inhibition of all motor activity as an alternative interpretation for these effects, they deemed rareness of errors as a trigger for these processes (e.g., Notebaert et al., 2009; Wessel & Aron, 2017). We prompted honest and dishonest responses in an equal frequency here, however.

Finally, prolonged responses after being dishonest might reflect a shift toward a more conservative response criterion. For errors, initial monitoring does indeed seem to be *followed* by such a shift in response threshold (Jentzsch & Dudschig, 2009; Steinhauser et al., 2017). That is, these criterion shifts seem to take time and would emerge rather for long that for short response–stimulus intervals. Instead, effects of dishonest responding on Task 2 vanished (Experiment 1) or became descriptively smaller (Experiment 2) with the long response–stimulus interval here. Furthermore, we observed a more liberal response criterion in an unrelated task after a modest response–stimulus interval of 400 ms between the dishonest response in a preceding study (Foerster et al., 2017a, 2017b).^1^ In a nutshell, we evaluate monitoring as the most feasible explanation for the observed effects considering all abovementioned findings from the literature.

### Integrating other aspects of lying

People can lie by very different means, i.e., they can deliver information that is incongruent with the truth, or they can simply withhold true information. Withholding an initially activated response also qualifies as a cognitive conflict supporting the notion that lying by omissions should be monitored as thoroughly as lying by commissions in the current study. Addressing this question in traditional psychological refractory period paradigms is not trivial, because these rely on the localization of effects in RTs. RTs are, however, not available for response omissions (for a discussion of the pitfalls in examining such non-actions, see Weller et al., 2017).

Traditional psychological refractory paradigms and their predictions also come to a limit when the aim is to examine effects in the postcentral, motor stage, while capacity-limited response monitoring processes are present and exceed response execution as in the current study (Foerster et al., 2019). One of the core assumptions is that the postcentral stage can operate in parallel with all stages of another task (Pashler, 1994). As such, effects in this stage do not propagate from Task 1 to Task 2. Capacity-limited processes operating until or after response execution would cover such effects in Task 1 making it impossible to detect them.

### Conclusions

The current experiments expand our knowledge of information processing in dishonest responding. Dishonest responses appear to be more thoroughly monitored than honest responses even past response execution. A prime candidate for the source of this extensive monitoring process is the parallel activation of both, honest and dishonest response tendencies. Although the employed paradigms aimed at an isolated examination of this conflict within dishonest responding, we cannot yet exclude that general response difficulty instead of conflict itself triggers extensive monitoring. Future examination should disentangle these aspects. We further recommend to explore monitoring of the effects of dishonest responses and its link to response monitoring.

### Electronic supplementary material

Below is the link to the electronic supplementary material.

Supplementary file1 (PDF 184 KB)

## Appendix

### Appendix A: Question sets

See Tables 1 and 2.

**Table 1:** Question set of Experiment 1 with German originals and English translations

Code	German original	English translation
1	Warst du Joggen?	Did you go for a run?
2	Bist du eine Treppe herunter gegangen?	Did you go down a staircase?
3	Bist du eine Treppe hoch gegangen?	Did you go up a staircase?
4	Hast du getankt?	Did you buy petrol?
5	Hast du Schokolade gegessen?	Did you eat chocolate?
6	Bist du Bus gefahren?	Did you take a bus?
7	Bist du Zug gefahren?	Did you take a train?
8	Hast du einen Mülleimer benutzt?	Did you use a dustbin?
9	Hast du ein Bad genommen?	Did you take a bath?
10	Hast du ein Toast zubereitet?	Did you make a sandwich?
11	Hast du einen Brief geschrieben?	Did you post a letter?
12	Hast du eine Tür geschlossen?	Did you close a door?
13	Warst du duschen?	Did you take a shower?
14	Hast du eine Zeitung gekauft?	Did you buy a newspaper?
15	Hast du eine Zeitschrift gekauft?	Did you buy a magazine?
16	Hast du ein Messer benutzt?	Did you use a knife?
17	Hast du einen Regenschirm benutzt?	Did you use an umbrella?
18	Hast du ein Medikament genommen?	Did you take a pill?
19	Hast du mit einem Polizisten gesprochen?	Did you speak to a police officer?
20	Hast du einen Apfel gegessen?	Did you eat an apple?
21	Hast du ein Fenster zerstört?	Did you break a window?
22	Hast du telefoniert?	Did you use a telephone?
23	Hast du eine SMS erhalten?	Did you receive a text?
24	Hast du einen Saft getrunken?	Did you drink fruit juice?
25	Hast du Radio gehört?	Did you listen to the radio?
26	Warst du im Internet?	Did you use the internet?
27	Hast du in einer Schlange angestanden?	Did you stand in a queue?
28	Hast du in einem Warteraum gesessen?	Did you sit in a waiting room?
29	Hast du dein Bett gemacht?	Did you make your bed?
30	Hast du deine Hände gewaschen?	Did you wash your hands?
31	Hast du ein Dokument unterzeichnet?	Did you sign a document?
32	Hast du Kaffee getrunken?	Did you drink coffee?
33	Hast du mit einem Kind gesprochen?	Did you speak to a child?
34	Hast du Fernsehen geschaut?	Did you watch television?
35	Hast du Zwiebeln gegessen?	Did you eat onions?
36	Hast du Wasser getrunken?	Did you drink water?
37	Hast du an einer Ampel gehalten?	Did you stop at a traffic light?
38	Warst du im Supermarkt?	Did you go to a supermarket?
39	Hast du Blumen gekauft?	Did you buy some flowers?
40	Hast du abgewaschen?	Did you do the dishes?
41	Bist du Fahrstuhl gefahren?	Did you take an elevator?
42	Hast du ein Fenster geputzt?	Did you clean a window?
43	Hast du eine Verabredung verschoben?	Did you reschedule an appointment?
44	Hast du ein Buch gelesen?	Did you read a book?
45	Hast du ein Moped abgestellt?	Did you park a moped?
46	Hast du eine Zitrone ausgepresst?	Did you squeeze a lemon?
47	Hast du eine Email verschickt?	Did you send an e-mail?
48	Hast du ein Tier gestreichelt?	Did you stroke a pet?
49	Hast du einen Mantel getragen?	Did you wear a coat?
50	Hast du einen Kühlschrank geöffnet?	Did you open a fridge?
51	Hast du einen Computer eingeschaltet?	Did you switch on a computer?
52	Hast du eine Zigarette geraucht?	Did you smoke a cigarette?
53	Hast du auf eine Uhr geschaut?	Did you look at a watch?
54	Hast du einen Wasserhahn geöffnet?	Did you open a water tap?
55	Hast du einen Toilettendeckel geöffnet?	Did you lift a toilet seat?
56	Bist du über einen Zebrastreifen gelaufen?	Did you use a pedestrian crossing?
57	Hast du einen Geldautomaten benutzt?	Did you use an ATM?
58	Hast du Geld gewechselt?	Did you change money?
59	Hast du einen Teppich abgesaugt?	Did you vacuum a carpet?
60	Hast du Hustensaft getrunken?	Did you drink cough syrup?
61	Hast du jemanden gegrüßt?	Did you greet someone?
62	Hast du geputzt?	Did you clean the house?
63	Hast du in deinen Briefkasten geschaut?	Did you check your mailbox?
64	Hast du deine Zähne geputzt?	Did you brush your teeth?
65	Hast du Musik gehört?	Did you listen to music?
66	Bist du Fahrrad gefahren?	Did you ride on a bicycle?
67	Hast du auf einer Leiter gestanden?	Did you stand on a ladder?
68	Hast du auf einem Stuhl gesessen?	Did you sit on a chair?
69	Hast du ein Stück Papier abgerissen?	Did you rip a piece of paper?
70	Hast du Blumen gegossen?	Did you water the plants?
71	Hast du deine Schlüssel benutzt?	Did you use your keys?
72	Hast du Wasser gekocht?	Did you boil some water?

**Table 2:** Question sets of Experiments 2 and 3 with German original and English translation. Explanations in brackets were not part of the question

Set	Code	German original	English translation
1	1	Hast du die Würfel gestapelt?	Did you stack the dice?
	2	Hast du die Bausteine getrennt?	Did you take apart the bricks?
	3	Hast du auf das Blatt gestempelt?	Did you stamp the piece of paper?
	4	Hast du die Stiftkappen vertauscht?	Did you swap the caps of the pens?
	5	Hast du in das Papiertuch getackert?	Did you staple the paper towel?
	6	Hast du die Klammer am Cent befestigt?	Did you clip the peg on the cent?
	7	Hast du den Kaffeefilter durchstochen?	Did you puncture the coffee filter?
	8	Hast du den Sticker auf den Teller geklebt?	Did you put the sticker on the plate?
	9	Hast du die Fliege [aus Papier] in die Schachtel gepackt?	Did you put the [paper] fly into the container?
	10	Hast du eine Schleife um die Gabel gebunden?	Did you tie a bow to the fork?
2	11	Hast du die Nudel zerbrochen?	Did you break the noodle?
	12	Hast du die Karte zerschnitten?	Did you cut the card?
	13	Hast du den Helikopter ausgemalt?	Did you color a helicopter?
	14	Hast du Reis in die Dose umgefüllt?	Did you decant the rice into the container?
	15	Hast du die Watte zur Kugel gerollt?	Did you form a ball from the cotton wool?
	16	Hast du den Draht vom Deckel entfernt?	Did you detach the wire from the cap?
	17	Hast du die Murmel in die Folie getan?	Did you put the marble into the transparent envelope?
	18	Hast du den Magneten aus der Kapsel geholt?	Did you take the magnet from the capsule?
	19	Hast du die Mutter von der Schraube gedreht?	Did you loosen the nut from the screw?
	20	Hast du Papier aus der Zeitschrift gerissen?	Did you rip paper from the magazine?

## Appendix B: Descriptive statistics

See Tables 3, 4, 5, 6, 7 and 8.

**Table 3:** Mean error rates in percent and response times (RTs) in ms and respective intention effects (Δ = dishonest – honest) with standard deviations (SDs) for each combination of error feedback, response–stimulus interval (RSI) and intention of the Intention Task 1 in Experiment 1

Error feedback	RSI	Intention	Mean error rate (SD)	Mean Δ error rate (SD)	Mean RT (SD)	Mean ΔRT (SD)
Early	0 ms	Honest	7.53 (5.5144)	7.35 (7.5854)	1052 (177.94)	155 (119.86)
		Dishonest	14.88 (9.1202)		1207 (243.18)	
	1000 ms	Honest	6.70 (4.9949)	8.11 (7.2802)	1084 (217.55)	142 (97.85)
		Dishonest	14.81 (9.0969)		1226 (254.06)	
Late	0 ms	Honest	7.95 (5.2491)	7.88 (8.1524)	1072 (177.06)	166 (141.13)
		Dishonest	15.83 (9.7712)		1238 (265.56)	
	1000 ms	Honest	8.66 (4.7351)	4.64 (6.2686)	1059 (171.56)	165 (120.24)
		Dishonest	13.30 (8.9168)		1224 (232.13)	

**Table 4:** Mean error rates in percent and response times (RTs) in ms and respective intention effects (Δ = dishonest – honest) with standard deviations (SDs) for each combination of error feedback, response–stimulus interval (RSI) and intention of the Tone Task 2 in Experiment 1

Error feedback	RSI	Intention	Mean error rate (SD)	Mean Δ error rate (SD)	Mean RT (SD)	Mean ΔRT (SD)
Early	0 ms	Honest	4.95 (3.9135)	0.44 (4.6663)	552 (81.50)	6 (27.51)
		Dishonest	5.39 (4.1786)		558 (84.13)	
	1000 ms	Honest	3.61 (3.8435)	−0.49 (2.8672)	443 (73.30)	−1 (22.48)
		Dishonest	3.12 (4.1928)		442 (76.77)	
Late	0 ms	Honest	6.24 (5.0732)	−1.48 (5.9072)	553 (87.38)	12 (31.84)
		Dishonest	4.77 (3.4078)		566 (86.55)	
	1000 ms	Honest	2.48 (2.3009)	−0.11 (2.6195)	432 (76.26)	−3 (29.77)
		Dishonest	2.37 (2.0711)		429 (82.87)	

**Table 5:** Mean error rates in percent and response times (RTs) in ms and respective intention effects (Δ = dishonest – honest) with standard deviations (SDs) for each combination of response–stimulus interval (RSI) and intention of the Intention Task 1 in Experiment 2

Error feedback	RSI	Intention	Mean error rate (SD)	Mean Δ error rate (SD)	Mean RT (SD)	Mean ΔRT (SD)
Late	0 ms	Honest	4.93 (6.0985)	7.27 (7.8314)	1291 (201.03)	142 (88.61)
		Dishonest	12.20 (8.3416)		1433 (236.63)	
	1000 ms	Honest	5.53 (5.9642)	5.82 (5.4544)	1306 (216.23)	129 (112.86)
		Dishonest	11.35 (6.9221)		1434 (229.65)	

**Table 6:** Mean error rates in percent and response times (RTs) in ms and respective intention effects (Δ = dishonest – honest) with standard deviations (SDs) for each combination of response–stimulus interval (RSI) and intention of the Letter Task 2 in Experiment 2

Error feedback	RSI	Intention	Mean error rate (SD)	Mean Δ error rate (SD)	Mean RT (SD)	Mean ΔRT (SD)
Late	0 ms	Honest	5.47 (4.7337)	0.59 (4.3202)	573 (69.08)	7 (26.23)
		Dishonest	6.06 (5.2020)		580 (78.84)	
	1000 ms	Honest	3.83 (4.1478)	−0.05 (3.7357)	473 (68.70)	5 (29.00)
		Dishonest	3.78 (3.9806)		478 (62.39)	

**Table 7:** Mean error rates in percent and response times (RTs) in ms and respective intention effects (Δ = dishonest – honest) with standard deviations (SDs) of the Intention Task 1 in Experiment 3

Error feedback	RSI	Intention	Mean error rate (SD)	Mean Δ error rate (SD)	Mean RT (SD)	Mean ΔRT (SD)
Late	0 ms	Honest	5.34 (3.6598)	6.80 (5.2161)	1390 (268.14)	150 (108.94)
		Dishonest	12.14 (5.8448)		1540 (288.58)	

**Table 8:** Mean error rates in percent and response times (RTs) in ms and respective intention effects (Δ = dishonest – honest) with standard deviations (SDs) of the Tone Task 2 in Experiment 3

Error feedback	RSI	Intention	Mean error rate (SD)	Mean Δ error rate (SD)	Mean RT (SD)	Mean ΔRT (SD)
Late	0 ms	Honest	8.20 (5.6187)	−0.06 (3.5046)	484 (68.29)	11 (24.04)
		Dishonest	8.15 (5.7561)		495 (72.40)	

## Footnotes

1 We additionally aimed for empirical evidence on this matter by demonstrating whether increased RT2 after dishonest responses maps to a delay of capacity-limited monitoring processes rather than to prolonged capacity-limited processing, which would point to a more conservative response threshold after dishonest responding instead. Therefore, we manipulated the difference in frequency of the to-be classified tones in Task 2, aiming for reduced perceptual discriminability and thus prolonged perceptual processing with smaller differences between tones. While monitoring would proceed in parallel with perceptual processes, predicting an interaction of (dis)honesty and perceptual difficulty in Task 2, a shift in response threshold should emerge independently because it would affect processes after perceptual processes have already finished. Although we replicated effects of extended monitoring of dishonest responding in Task 2, our manipulation was not successful in manipulating the perceptual stage in the first place, suggesting that an empirical distinction between both accounts might require different methodology (see the Supplementary Material for a summary of the findings).
