# Supplementary material for: Post-execution monitoring in dishonesty
Source: Psychol Res. 2022 Jun 25;87(3):845–61. doi: 10.1007/s00426-022-01691-x (PMC10017645; doi:10.1007/s00426-022-01691-x)
Supplement: Supplementary file 1 — Supplementary file1 (PDF 184 KB) [file 426_2022_1691_MOESM1_ESM.pdf]

Post-execution monitoring in dishonesty

Follow-up experiments

Anna Foerster, Roland Pfister, Robert Wirth, & Wilfried Kunde

University of Würzburg, Department of Psychology III

1 Background: Experiments 1-3

Please first consult the main text about the initial three experiments of this project for a thorough theoretical and empirical background. We conducted two additional experiments to examine whether previous effects of a dishonest response on the immediately following response indeed map to capacity-limited monitoring (Experiment 4-5). We preregistered both experiments (Experiment 4: osf.io/t9w6h, Experiment 5: osf.io/cnj74) and uploaded their materials, data and analyses scripts to the OSF repository of the original three experiments (osf.io/7axw9).

2 Experiment 4

The former experiments of this project demonstrated that dishonest compared to honest responding in a Task 1 delays responding in an immediately following unrelated Task 2. We attributed this effect to prolonged capacity-limited monitoring after dishonest responses that delayed central capacity-limited response selection processes in Task 2 (Foerster et al., 2019; Jentzsch & Dudschig, 2009; Jentzsch et al., 2007; Steinhauser et al., 2017). Alternatively, dishonest responding might have quickly instantiated a more conservative response criterion that was already effective in an immediately following Task 2, prolonging (instead of delaying) central processing of Task 2 (e.g., Jentzsch & Dudschig, 2009). We aimed for a variation of the extent of perceptual processing in Task 2 to disentangle these two alternatives because perceptual processes are typically assumed to run in parallel with capacity-limited processes of another task (e.g., Pashler, 1994; Jentzsch et al., 2007). Accordingly, prolonging perceptual processing should allow monitoring processes to finish before subsequent central processes are due. Therefore, we expected an interaction between (dis)honesty and perceptual difficulty in Task 2, with a smaller impact of dishonesty with greater perceptual difficulty. In contrast, prolonged central processing of Task 2 due to a more conservative response

threshold after dishonest responding in Task 1 would emerge independently of perceptual difficulty, because central processing would only start upon finishing precentral processing. [1]

We aimed to disentangle these alternatives in a setup similar to the experiments described in the main manuscript. Participants conducted a (Dis)honest Task 1 and a Tone Task 2 in close succession. Crucially, we manipulated between experimental blocks whether there was a large or a small difference in frequency between the two to-be classified tones (300 Hz and 800 Hz vs. 500 Hz and 600 Hz), assuming greater perceptual difficulty for small differences. If dishonest responding in Task 1 triggers prolonged post-response capacity-limited monitoring, delaying capacity-limited central processing of Task 1, an interaction between intention (honest vs. dishonest) and tone difference (100 Hz vs. 500 Hz) should emerge in Task 2, with smaller monitoring costs for 100 Hz tone differences. In contrast, we should find additive effects of Task 1 intention and Task 2 tone discrimination difficulty if dishonest responding in Task 1 prolongs the capacity-limited central stage of Task 2.

2.1 Methods

We describe methodological aspects where Experiment 4 deviated from Experiment 3 of the main article for brevity.

Participants

We considered effect sizes of the former experiment where we also used tones in Task 2 and employed a late feedback condition as in the current experiment (Experiment 1: d_z = 0.39 and Experiment 3[2]: d_z = 0.44). These effect sizes emerged in trials where the tones were equivalent to the current 500 Hz tone difference condition. For the 100 Hz tone differences employed here, the intention effect might vanish entirely. Therefore, we approximated an estimate of the effect size of the interaction of intention and tone difference by pitting the formerly obtained intention effects against 0. Following this logic, we expected a similar effect size for the interaction as for intention effects themselves in the former experiments. As such, we opted for 64 participants in our statistical analyses.

[1] Alternatively, similar additive effects should emerge if we assume a postponement of all processes, including precentral processes, of Task 2 until any monitoring processes of Task 1 have finished (see Wirth et al., 2017).

[2] There was a typo in the preregistration that linked this effect size falsely to Experiment 2 where we used letter stimuli in Task 2.

This sample size provides a power of 87% with an alpha of 5% to detect an intention effect of d_z = 0.39 in a two-sided test (calculated with the power.t.test function in R version 3.3.3).

Seventy-six participants (mean age = 26 [SD = 9.64] years) took part in the study. We had to exclude twelve participants because they responded correctly in less than 60% of the trials, failing to meet our preregistered inclusion criterion. Seventeen participants self-identified as male, 59 as female, and eight identified as left-handed. All participants provided written informed consent and received monetary compensation or course credit.

In the analysis of the error rates of Task 2, we noticed that one participant performed at chance level in blocks with a 100 Hz difference between tones (50% vs. 11% commission errors in the 100 Hz and 500 Hz condition, respectively). We did not anticipate that participants might fail completely to discriminate the tones in the preregistration. However, this participant was clearly not able to conduct the task properly in the 100 Hz condition. We therefore excluded this participant without replacement.

Apparatus, stimuli and procedure

In alternating blocks, we presented either two tones with a small difference (500 Hz and 600 Hz) or with a large difference (300 Hz and 800 Hz) in frequency. We counterbalanced whether participants started with a block with a small or a large tone difference. The combination of 20 questions × 2 intentions (honest vs. dishonest) × 2 tones (300 Hz vs. 800 Hz / 500 Hz vs. 600 Hz) resulted in 80 different trials per block. Participants went through eight blocks of these randomized trials with self-paced breaks after each 40th trial. Half of the blocks featured tones with a 100 Hz tone difference and the other half tones with a 500 Hz tone difference, and participants alternated between both block types.

2.2 Results

Data treatment

We eliminated practice trials from the data and the first trial after each self-paced break. We further excluded post-error trials (19.3%). For the error rate analysis of the Intention Task 1, we excluded premature responses (0.1%) and omission errors (0.9%) in this task. For the error rate analysis of the Tone Task 2, we considered trials with a correct response in Task 1 and excluded response omissions in the Tone Task 2 (1.7%). For RT analyses of both tasks, we selected trials with

correct responses in both tasks. We further excluded trials where at least one response qualified as an outlier (4.4%). All participants delivered at least 10 observations in each cell after these exclusions and could thus be included in the following statistical analyses.

Analyses plan

Table 1 and 2 in Appendix B provide an overview of the descriptive statistics. Figure 1 depicts mean RTs. We analyzed error rates and RTs in analyses of variance (ANOVAs) with the within-subjects factors intention (honest vs. dishonest) and tone difference (100 Hz vs. 500 Hz). We followed up on significant two-way interactions in planned one-tailed paired-samples *t*-tests.

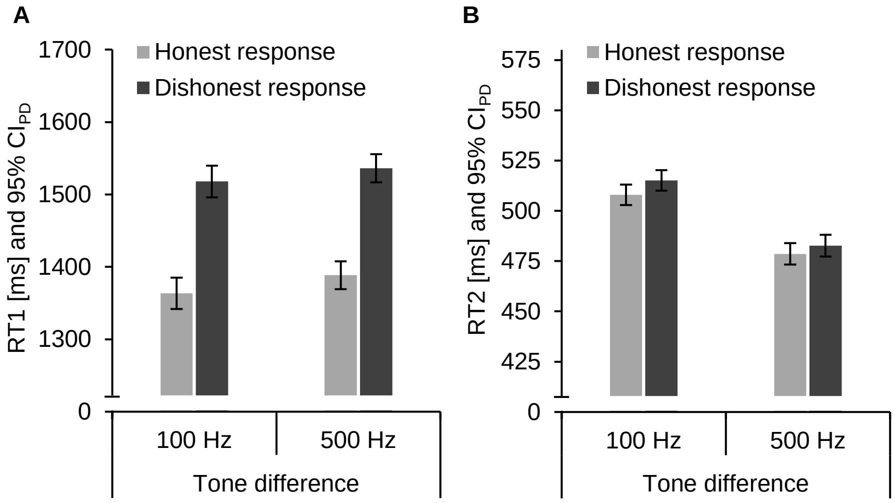

Figure 1. Mean response times (RTs) of the Intention Task 1 (A) and the Tone Task 2 (B) of honest (light grey) and dishonest (dark grey) responses in Experiment 4. Error bars depict 95% confidence intervals of paired differences (CI_{PD}), computed separately for each tone difference.

Intention Task (Task 1)

Responding was prolonged for dishonest compared to honest responses, $F(1, 62) = 258.08$, $p < .001$, $\eta_p^2 = .81$, and in blocks with 500 Hz tone differences compared to blocks with 100 Hz differences, $F(1, 62) = 6.44$, $p = .014$, $\eta_p^2 = .09$. The interaction of both factors was not significant, $F < 1$.

Dishonest responses came with higher error rates than honest responses, $F(1, 62) = 94.79$, $p < .001$, $\eta_p^2 = .61$. In blocks with 100 Hz compared to 500 Hz tone differences, participants also

committed more errors in the Intention Task 1, $F(1, 62) = 6.47$, $p = .013$, $\eta_p^2 = .10$. The interaction between both factors was not significant, $F(1, 62) = 3.58$, $p = .063$, $\eta_p^2 = .06$.

Tone Task (Task 2)

Responding in the Tone Task 2 was slower after a dishonest than after an honest response in the Intention Task 1, $F(1, 62) = 6.60$, $p = .013$, $\eta_p^2 = .10$. Tone differences of 100 Hz prolonged responses compared to 500 Hz tone differences, $F(1, 62) = 65.29$, $p < .001$, $\eta_p^2 = .51$. The interaction of both factors was not significant, $F(1, 62) = 1.15$, $p = .287$, $\eta_p^2 = .02$.

Error rates were lower after honest than after dishonest responses in Task 1, $F(1, 62) = 6.35$, $p = .014$, $\eta_p^2 = .09$, and for 500 Hz than for 100 Hz tone differences, $F(1, 62) = 31.10$, $p < .001$, $\eta_p^2 = .33$. The interaction was not significant, $F(1, 62) = 1.49$, $p = .227$, $\eta_p^2 = .02$.

2.3 Discussion

In the current experiment, we manipulated between blocks how strongly the to-be-classified tones in Task 2 differed in frequency. We hypothesized that this manipulation would affect the length of precentral processing and that prolonged monitoring of dishonest responses would proceed in parallel with extended precentral processing. Instead, prolonged central processing in Task 2 because of a more conservative response criterion after dishonest responses should emerge independently of any precentral processing length. We indeed found additive effects of intention and tone difference in the Tone Task 2, supporting the assumption of a shift to a more conservative response threshold after dishonest responding. There is one alternative interpretation for these results though: Our manipulation of tone differences might not have succeeded in prolonging precentral processing but instead might have altered central processing of the Tone Task 2 and we tested this possibility in Experiment 5.

3 Experiment 5

To test whether the effect of tone differences on the Tone Task 2 in Experiment 4 originated in processes of the precentral or central stage, we adopted the prominent locus-of-slack logic (e.g., Pashler, 1994). According to this logic, Task 2 becomes slower with an increasing overlap between the two tasks because the central processing stage of Task 2 only proceeds when central processing of Task 1 has ended. This waiting time is referred to as cognitive slack. Crucially, precentral processes

of Task 2 can run in parallel with any Task 1 processes. As such, a stronger overlap between tasks would enable precentral processing of Task 2 to proceed during the cognitive slack, leading to the prediction that a manipulation of the precentral stage should have a smaller impact with a strong than with a weaker overlap between tasks.

We employed a simpler Task 1 with a classification of two different colors via keypresses. The Color Task 1 and the Tone Task 2 overlapped with a stimulus-onset asynchrony between stimuli of both tasks of either 0 ms or 500 ms. We again manipulated between blocks whether tone differences were 100 Hz or 500 Hz as in the preceding experiment. If effects of tone differences map to precentral processes, stimulus-onset asynchrony and tone discrimination should interact in Task 2, with larger effects of tone differences for the 500 ms than the 0 ms stimulus-onset asynchrony. In contrast, if the effect of tone differences maps to the capacity-limited central stage of Task 2, effects of stimulus-onset asynchrony and tone differences should be additive.

3.1 Methods

We keep this section brief by reporting only aspects where Experiment 5 deviated from Experiment 4.

Participants

The effect of tone differences in Task 2 of Experiment 4 amounted to $d_z = 1.02$ in response times (RTs). As a best-case scenario, an effect of similar size would emerge for the 500 ms stimulus-onset asynchrony and would vanish entirely for the 0 ms stimulus-onset asynchrony. We could thus approximate an estimate of the effect size of the interaction of stimulus-onset asynchrony and tone discrimination by pitting the formerly obtained tone discrimination effect against 0. Following this logic, we would expect a similar effect size for the interaction as for the tone difference effect of the former experiment. A sample size of about 15 participants provides a power of 95% with an alpha of 5% to detect an effect of $d_z = 1.02$ in a two-tailed test (calculated with the power.t.test function in R version 3.3.3). Considering our counterbalancing factors, we decided to include a sample size of 16 participants in our statistical analyses.

We collected data of 17 participants (mean age = 28 [SD = 7.73] years) because one participant responded correctly in less than 60% of the trials and was therefore replaced. Four participants self-identified as male and nine participants as left-handed.

Apparatus, stimuli and procedure

In the Color Task 1, participants classified colored squares (blue vs. yellow) via the response keys *D* and *F*. We sticked corresponding color patches on the response keys. We counterbalanced the assignment of colors to keys across participants. The stimulus-onset asynchrony of color patch and tone was 0 ms or 500 ms, in a random sequence.

The combination of 2 stimulus-onset asynchronies (0 ms vs. 500 ms) × 2 colors (yellow vs. blue) × 2 tones (300 Hz vs. 800 Hz / 500 Hz vs. 600 Hz) resulted in eight individual trial combinations, which we presented five times in a random order in each block. Participants went through two practice blocks and sixteen experimental blocks of these randomized trials with self-paced breaks in between. Half of the blocks featured tones with a 100 Hz tone difference and the other half tones with a 500 Hz tone difference, and participants alternated between both block types after every second experimental block (AABB design).

3.2 Results

Data treatment

We excluded practice trials from the data and the first trial after each self-paced break. We only selected trials with a correct response in the preceding trial (9.9% excluded). For the error rate analysis of the Color Task 1, we excluded premature responses (< 0.1%) and omission errors (0.1%) in this task. For the error rate analysis of the Tone Task 2, we selected trials with a correct response in Task 1 and excluded premature responses before stimulus onset (< 0.1%) and response omissions (0.2%) in the Tone Task 2. For RT analyses of both tasks, we selected trials with correct responses in both tasks. We further excluded trials where the interval between responses of the two tasks was smaller than 100 ms (3.7%) to control for potential effects of grouped responses and we eliminated outlier trials (3.7%). All participants delivered at least 10 observations in each cell after these exclusions and could thus be included in the following statistical analyses.

Analyses plan

Detailed descriptive statistics are provided in Table 3 and 4 in Appendix B and Figure 2 shows mean RTs. We analyzed error rates and RTs of the Color Task 1 and the Tone Task 2 in separate ANOVAs with the within-subjects factors stimulus-onset asynchrony (0 ms vs. 500 ms) and tone difference (100 Hz vs. 500 Hz). We scrutinized significant two-way interactions in planned two-tailed paired-samples *t*-tests.

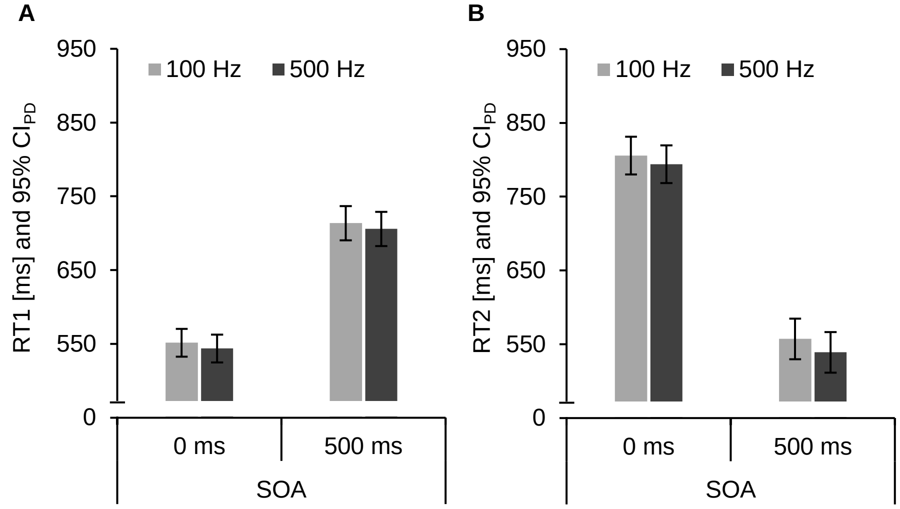

Figure 2. Mean response times (RTs) of the Color Task 1 (A) and the Tone Task 2 (B) of blocks with a 100 Hz (light grey) and a 500 Hz (dark grey) difference between tones in Experiment 5. Error bars depict 95% confidence intervals of paired differences (CI_{PD}), computed separately for stimulus-onset asynchrony (SOA).

Color Task (Task 1)

RTs in the Color Task 1 were prolonged for the 500 ms relative to the 0 ms stimulus-onset asynchrony, $F(1, 15) = 30.90$, $p < .001$, $\eta_p^2 = .67$, whereas the other effects were not significant, *F*s < 1. Error rates were higher with a stimulus-onset asynchrony of 0 ms than 500 ms, $F(1, 15) = 11.41$, $p = .004$, $\eta_p^2 = .43$. The main effect of tone difference and the two-way interaction were not significant, *F*s(1, 15) ≤ 1.28, *p*s ≥ .276, $\eta_p^2 \leq .08$.

Tone Task (Task 2)

RTs in the Tone Task 2 were lower with the 500 ms stimulus-onset asynchrony than with the 0 ms stimulus-onset asynchrony, $F(1, 15) = 462.66$, $p < .001$, $\eta_p^2 = .97$. The main effect of tone

difference and the interaction were not significant, *F*s(1, 15) ≤ 1.99, *p*s ≥ .179, η_p^2 ≤ .12. Error rates were larger for 100 Hz than 500 Hz tone differences, *F*(1, 15) = 12.07, *p* = .003, η_p^2 = .45. The main effect of stimulus-onset asynchrony and the interaction were not significant, *F*s < 1.

3.3 Discussion

In this experiment, we tested whether the manipulation of the differences between tones in Task 2 maps to the precentral processing stage. Therefore, we manipulated the stimulus-onset asynchrony of a Color Task 1 and a Tone Task 2, hypothesizing that the effect of tone difference in the Tone Task 2 would be smaller for the short than the long stimulus-onset asynchrony. However, the expected effect of tone difference only emerged in error rates, across SOAs. As such, the manipulation of the precentral stage through tone differences was probably also not successful in Experiment 4, precluding any inferences about the nature of post-response processes after dishonest actions from these results. The approach of manipulating tone differences between blocks of trials might have provoked strategic adaptations of central processing, suggesting that an empirical distinction between extended monitoring and prolonged central processing is difficult to achieve.

4 References

Foerster, A., Wirth, R., Berghoefer, F. L., Kunde, W., & Pfister, R. (2019). Capacity limitations of dishonesty. *Journal of Experimental Psychology: General*, *148*(6), 943-961 http://dx.doi.org/10.1037/xge0000510

Jentzsch, I., & Dudschig, C. (2009). Why do we slow down after an error? Mechanisms underlying the effects of posterror slowing. *The Quarterly Journal of Experimental Psychology, 62*(2), 209-218. http://dx.doi.org/10.1080/17470210802240655

Jentzsch, I., Leuthold, H., & Ulrich, R. (2007). Decomposing sources of response slowing in the PRP paradigm. *Journal of Experimental Psychology: Human Perception and Performance, 33*(3), 610-626. http://dx.doi.org/10.1037/0096-1523.33.3.610

Pashler, H. (1994). Dual-task interference in simple tasks: Data and theory. *Psychological Bulletin, 116*(2), 220-244. http://dx.doi.org/10.1037/0033-2909.116.2.220

Steinhauser, M., Ernst, B., & Ibald, K. W. (2017). Isolating component processes of posterror slowing with the psychological refractory period paradigm. *Journal of Experimental Psychology: Learning, Memory, and Cognition, 43(*4), 653-659. http://dx.doi.org/10.1037/xlm0000329

Wirth, R., Janczyk, M., & Kunde, W. (2018). Effect monitoring in dual-task performance. *Journal of Experimental Psychology: Learning, Memory, and Cognition, 44*(4), 553-571. http://dx.doi.org/10.1037/xlm0000474

5 Appendices

Table 1. Mean error rates in percent and response times (RTs) in ms and respective intention effects (Δ = dishonest – honest) with standard deviations (SDs) for each combination of tone difference and intention of the Intention Task 1 in Experiment 4.

Error feedback	RSI	Tone difference	Intention	Mean error rate (SD)	Mean Δ error rate (SD)	Mean RT (SD)	Mean ΔRT (SD)
Late	0 ms	100 Hz	Honest	4.34 (3.4110)	6.69 (5.9065)	1363 (191.87)	155 (86.63)
			Dishonest	11.03 (7.3674)		1518 (218.65)	
		500 Hz	Honest	4.00 (2.6552)	5.60 (5.0827)	1389 (203.43)	148 (76.79)
			Dishonest	9.60 (6.2931)		1536 (226.45)	

Table 2. Mean error rates in percent and response times (RTs) in ms and respective intention effects (Δ = dishonest – honest) with standard deviations (SDs) for each combination of tone difference and intention of the Tone Task 2 in Experiment 4.

Error feedback	RSI	Tone difference	Intention	Mean error rate (SD)	Mean Δ error rate (SD)	Mean RT (SD)	Mean ΔRT (SD)
Late	0 ms	100 Hz	Honest	7.71 (5.1658)	1.32 (4.1075)	508 (73.91)	7 (20.36)
			Dishonest	9.03 (6.5414)		515 (78.71)	
		500 Hz	Honest	5.29 (3.8239)	0.56 (3.6169)	479 (77.39)	4 (21.25)
			Dishonest	5.86 (4.042)		483 (85.58)	

Table 3. Mean error rates in percent and response times (RTs) in ms and respective tone difference effects (Δ = 100 Hz – 500 Hz) with standard deviations (SDs) for each combination of stimulus-onset asynchrony (SOA) and tone difference of the Color Task 1 in Experiment 5.

Error feedback	SOA	Tone difference	Mean error rate (SD)	Mean Δ error rate (SD)	Mean RT (SD)	Mean ΔRT (SD)
Late	0 ms	100 Hz	3.62 (3.0236)	-0.43 (2.2439)	552 (94.48)	8 (35.49)
		500 Hz	4.05 (2.8831)		544 (107.30)	
	500 ms	100 Hz	1.89 (1.9252)	-0.37 (1.3476)	714 (203.15)	8 (43.56)
		500 Hz	2.26 (2.3159)		706 (212.94)	

Table 4. Mean error rates in percent and response times (RTs) in ms and respective tone difference effects (Δ = 100 Hz – 500 Hz) with standard deviations (SDs) for each combination of stimulus-onset asynchrony (SOA) and tone difference of the Tone Task 2 in Experiment 5.

Error feedback	SOA	Tone difference	Mean error rate (SD)	Mean Δ error rate (SD)	Mean RT (SD)	Mean ΔRT (SD)
Late	0 ms	100 Hz	7.58 (4.6172)	3.58 (5.6376)	806 (90.90)	12 (48.03)
		500 Hz	4.00 (2.8048)		794 (100.14)	
	500 ms	100 Hz	8.47 (5.1790)	4.50 (4.8264)	557 (117.86)	18 (51.86)
		500 Hz	3.97 (2.8561)		539 (125.95)	
